# Prognostic Factors in Newly Diagnosed High‐Grade Osteosarcoma—A Systematic Review

**DOI:** 10.1002/cam4.71044

**Published:** 2025-07-16

**Authors:** Elisa Tirtei, Sascha Wilk Michelsen, Lianne M. Haveman, Cristina Meazza, Joana F. Oliveira, Ayesha Rasool, Emanuela Palmerini, Will Wilson, Nathalie Gaspar, Sandra J. Strauss, Andri Papakonstantinou, Fredrik Baecklund, the FOSTER Consortium (Fight OsteoSarcoma Through European Research), work package 4 on trials in newly diagnosed osteosarcoma

**Affiliations:** ^1^ Department of Pediatric Oncology Regina Margherita Children's Hospital Turin Italy; ^2^ Department of Pediatric and Adolescence Medicine, Clinic for Pediatric Oncology and Hematology University Hospital Rigshospitalet Copenhagen Denmark; ^3^ Princess Maxima Center for Pediatric Oncology Utrecht the Netherlands; ^4^ Pediatric Oncology Unit Fondazione IRCCS Istituto Nazionale dei Tumori Milan Italy; ^5^ Pediatric Department Portuguese Institute of Oncology of Lisbon Lisbon Portugal; ^6^ UCL Cancer Institute London UK; ^7^ Osteoncology, Bone and Soft Tissue Tumors and Innovative Therapies IRCCS Istituto Ortopedico Rizzoli Bologna Italy; ^8^ Miller School of Medicine University of Miami Miami Florida USA; ^9^ Cancer Research UK and UCL Cancer Trials Centre London UK; ^10^ Department of Oncology for Child and Adolescent Gustave Roussy Cancer Campus Villejuif France; ^11^ Department of Oncology UCL Cancer Institute London UK; ^12^ Department of Oncology‐Pathology Karolinska Institutet Stockholm Sweden; ^13^ Department of Breast Cancer, Endocrine Tumors and Sarcoma Karolinska Comprehensive Cancer Center, Karolinska University Hospital Stockholm Sweden; ^14^ Pediatric Oncology Karolinska University Hospital Stockholm Sweden; ^15^ Department of Molecular Medicine and Surgery, Rare Disease Group Karolinska Institutet Stockholm Sweden

**Keywords:** newly diagnosed, osteosarcoma, pretreatment, prognostic factors, systematic review

## Abstract

**Introduction:**

Pretreatment prognostic factors in newly diagnosed osteosarcoma are important for clinical management and stratifying patients in clinical trials. Such factors include the presence of metastases, primary tumor size, and site. Factors surrounded by controversy include pathological fracture, histologic subtype, and P‐glycoprotein expression. No prognostic tumor biomarker has been established. We performed a systematic review with the aim to compile available evidence for pretreatment prognostic factors and define optimal cut‐off values for patient stratification or further validation in the upcoming European FOSTER‐CabOS trial.

**Methods:**

Predefined search terms were used to search PubMed, Web‐of‐science, and Embase for all studies investigating pretreatment prognostic factors in newly diagnosed osteosarcoma patients published 2000–2023. After applying strict inclusion and exclusion criteria, 49 papers were included.

**Results:**

We found 14 factors investigated in at least two separate studies or in a single study using one discovery and at least one validation cohort.

**Conclusions:**

We confirmed the prognostic value of patient age, presence of metastasis, tumor size, and site (axial vs. appendicular). Future studies of these factors should focus on specific patient populations and defining optimal cut‐off values. Although serum level of alkaline phosphatase and lactate dehydrogenase were associated with outcome, it remains unclear if they are independent of other prognostic factors. The prognostic value remains unclear for sex, pathological fracture, histologic subtype, and P‐glycoprotein expression. We could not establish any new prognostic biomarker. However, circulating tumor DNA in plasma and the G1/G2 RNA signature in diagnostic tumor biopsies show promise and will be further validated in the upcoming FOSTER‐CabOS trial.

**Abbreviations**
ALP: alkaline phosphatase
CSS: cause‐specific survival
ctDNA: circulating tumor DNA
DSS: disease‐specific survival
EFS: event‐free survival
LDH: lactate dehydrogenase
MFS: metastases‐free survival
NLR: neutrophil‐to‐lymphocyte ratio
OS: overall survival
PgP: P‐glycoprotein

## 1 Introduction

Osteosarcoma is a rare malignant tumor of the bone [1]. Although rare, osteosarcoma is the most common bone sarcoma in children, adolescents, and young adults [2]. Treatment with complete tumor resection and chemotherapy results in approximately 70% 5‐year overall survival (OS) [2, 3, 4]. Despite intensive treatment, 30%–35% of patients experience disease recurrence associated with poor prognosis [1, 5, 6].

It is important to be able to identify patients with different prognoses already at diagnosis, before starting any treatment, for adequate clinical management and for stratifying patients in clinical trials. Currently, established pretreatment prognostic factors are the presence of metastases, primary tumor size, and non‐extremity site (axial and pelvic) [2, 7, 8]. No prognostic tumor biomarker has been established to date.

Previous attempts to stratify up‐front treatment by favorable (e.g., small tumor size) and unfavorable (e.g., presence of metastases or axial location) prognostic factors have failed to improve outcomes [9, 10]. Nevertheless, future osteosarcoma treatment needs to be tailored by prognostic and predictive baseline markers to improve the outcome of osteosarcoma patients. Thus, establishing robust prognostic factors present at diagnosis is an unmet need for future trials and clinical management. Preclinical and clinical research is essential to advance the field.

For the upcoming European FOSTER‐CabOS trial (EU CT number 2023‐505575‐69‐00), we conducted a systematic review to identify all studies assessing prognostic factors in newly diagnosed osteosarcoma published between 2000 and 2023, with the aim to compile all available evidence on established prognostic factors and identify new biomarkers with prognostic properties to use for patient stratification or further validation.

## 2 Methods

A comprehensive search for scientific papers investigating prognostic factors in newly diagnosed osteosarcoma was performed according to the Preferred Reporting Items for Systematic Review and Meta‐Analysis criteria (PRISMA 2020) [11]. PubMed (https://pubmed.ncbi.nlm.nih.gov/), Embase (https://www.embase.com), and Web of Science (https://www.webofscience.com) were searched for relevant studies utilizing the following key terms: “osteosarcoma” OR “osteogenic sarcoma” OR “osteosarcoma tumor” AND “prognos*” OR “predict*” OR “risk*” OR “stratif*”.

Inclusion and exclusion criteria used to select appropriate studies are listed in Table 1. In the first step, study selection and quality appraisal were performed by five reviewers (F.B., E.T., A.P., L.M.H., S.W.M.) independently and in duplicate. Titles and abstracts were screened to identify potentially relevant articles and to exclude those that clearly did not fit the scope of this review. When two reviewers disagreed on an identified study, a third reviewer made an assessment, and his/her decision was decisive. As a second step, the full texts of the selected articles were assessed by seven reviewers independently (F.B., E.T., A.P., L.M.H., C.M., S.W.M., J.F.O.) for eligibility based on the inclusion and exclusion criteria. Studies were also excluded if their scientific quality could not be adequately assessed, that is, if details were lacking to clearly define the study population, prognostic and confounding factors and outcome measurements, and statistical methods used.

**TABLE 1:** Inclusion and exclusion criteria list for this systematic search.

Inclusion criteria	Exclusion criteria
Studies on humans High‐grade osteosarcoma Studies at first diagnosis Studies investigating factors at baseline (before treatment) Any histologic type and stage Any publication of original data (clinical trial, retrospective trial, real world data) Case series with at least 10 patients will be admitted Time of publication: January 2000—December 2023 English language	Osteosarcoma as second malignancy Low‐grade osteosarcoma Relapsed osteosarcoma Prognostic factors measured after start of treatment Preclinical studies Papers with low quality (poor data description, no statistical analyses reported) precluding assessment of scientific quality Papers with only univariate analysis of the prognostic factor Studies assessing a factor that was not validated in an independent patient cohort (either another study or a validation cohort)

For studies applicable to the inclusion and exclusion criteria, and with adequate scientific quality, key variables were collected, including study type (prospective/retrospective, multi/single center), total number of patients, age range, primary treatment, prognostic factor evaluated, strata and number of patients in each stratum, outcome measures used, point estimate with 95% confidence interval (CI) and *p* value, and statistical methods used (univariate/multivariate, type of regression model, covariates). When studies reported two outcome measures, we collected and presented data for both.

Prognostic factors investigated in at least two studies or investigated in one study that used a discovery cohort and at least one validation cohort were included, whereas prognostic factors that were not validated in an independent cohort were excluded.

Collected data were summarized in tables and ordered by type of prognostic factor assessed and strength of evidence, defined by study design (prospective/retrospective) and sample size (larger number of study participants were rated higher than smaller numbers).

## 3 Results

**FIGURE 1:**
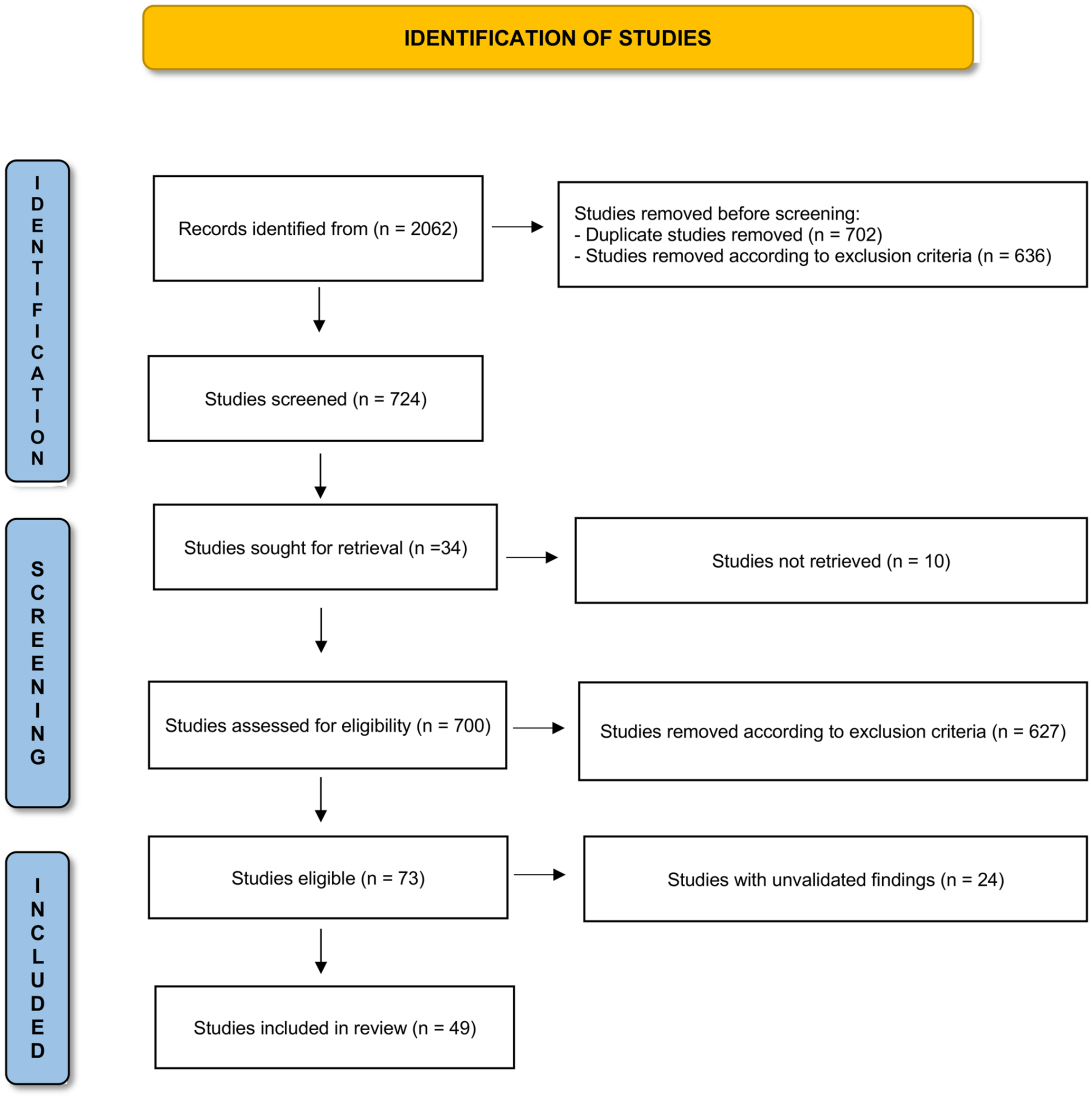
The PRISMA flow diagram visually represents the study selection process.

Forty‐nine studies remained after the selection process was completed (Figure 1). Fourteen pretreatment prognostic factors in newly diagnosed osteosarcoma were identified and categorized into three main groups: patient features, tumor features, and serum and plasma markers.

Overall, there was heterogeneity between study populations in terms of age range, disease extent (local, distant, both), and primary treatment given. Furthermore, different outcome measures were used, including OS and several surrogate endpoints: event‐free survival (EFS), cause‐specific survival (CSS), cancer‐specific survival, disease‐specific survival (DSS), progression‐free survival (PFS), metastasis‐free survival (MFS), relapse‐free survival (RFS), and lung metastasis‐free survival.

### 3.1 Patient Features

#### 3.1.1 Age at Diagnosis

Age at diagnosis was assessed in nine studies (Table 2), five multicenter (two prospective [2, 12], two retrospective [15, 17], one register‐based [16]) and four single‐center studies (two prospective) [13, 14, 18, 19]. The median number of patients included was 967 (range: 288–3435). The cut‐off values and number of age categories used (two, three or four) differed between studies. In all studies except one [19], the risk of an adverse outcome increased with increasing age and was highest for patients > 60 years at diagnosis. Associations were statistically significant for all surrogate endpoints and for seven out of ten associations with OS [2, 18].

**TABLE 2:** Studies of patient features as prognostic factors in newly diagnosed osteosarcoma. The studies are organized by prospective/retrospective data collection, prognostic factor categorization, reference value used, and number of included patients (*N*).

First author	Journal	Year	Multi/single center	Data collected prospectively/retrospectively	Population	*N*	Age span	Primary treatment	Strata	*N* per strata	Surrogate endpoints				Overall survival			Variables in multivariate model	Ref
												Point estimate	95% confidence interval	*p*	Point estimate	95% confidence interval	*p*		
*Age at diagnosis*																			
Smeland	European Journal of Cancer	2019	Multi center	Prospective	Local and metastatic	2186	< 40	MAP+/−IFNα/MAPIE	Child (ref)	557	Event‐free survival	Ref			Ref			A	[2]
									Adolescent	921		HR = 1.25	1.05–1.48	0.01	HR = 1.32	1.06–1.65	0.014		
									Adult	389		HR = 1.32	1.07–1.63	0.008	HR = 1.27	0.97–1.66	0.081		
Tian S	Translational Oncology	2022	Multi center	Prospective	Local and metastatic	1199	Median 17, IQR 12–28	Not reported	< 20 (ref)	525	Cancer‐specific survival	Ref			Not reported			B	[12]
									20–45	207		HR = 1.89	1.41–2.52	< 0.001	Not reported				
									≥ 45 years	108		HR = 3.69	2.69–5.05	< 0.001	Not reported				
Bacci	Cancer	2006	Single center	Prospective	Localized	783	All ages	MAP/MAPBCD/MAPI/MAPIE	> 14 (ref) versus ≤ 14 years	457/326	Event‐free survival	HR = 1.3	1.0–1.7	0.044	Not reported			C	[13]
Ferrari	Annals of Oncology	2001	Single center	Prospective	Localized	300	< 40	MAP/MAPI	> 12 (ref) versus ≤ 12 years	71/229	Disease‐specific survival	HR = 1.7	1.1–2.6	0.01	Not reported			D	[14]
Ottesen	JAAOS: Global Research & Reviews	2022	Multi center	Retrospective	Appendicular, local and metastatic	3435	< 23 to 62+	Not specified	< 23 years (ref)	Not reported		Not reported			Ref			Not specified	[15]
									23–45 years	Not reported					HR = 1.50	1.09–2.05	< 0.01		
									46–62 years	Not reported					HR = 2.24	1.61–3.12	< 0.001		
									> 62 years	Not reported					HR = 4.09	2.78–6.02	< 0.001		
Ottesen	JAAOS: Global Research & Reviews	2022	Multi center	Retrospective	Axial, local and metastatic	810	< 23 to 62+	Not specified	< 23 years (ref)	Not reported		Not reported			Ref			Not specified	[15]
									23–45 years	Not reported					HR = 1.33	1.14–1.55	< 0.001		
									46–62 years	Not reported					HR = 2.11	1.73–2.56	< 0.001		
									> 62 years	Not reported					HR = 3.53	2.70–4.61	< 0.001		
Duchman	Cancer Epidemiology	2015	Register based	Retrospective	Local and metastatic	2849	All ages	Not reported	0–24 (ref)	1825	Cause‐specific survival	Ref			Not reported			E	[16]
									25–59	676		HR = 1.5	1.30–1.79	< 0.05					
									≥ 60 years	348		HR = 2.8	2.30–3.46	< 0.05					
Fukushima	BMC Musculoskeletal Disorders	2018	Multi center	Retrospective	Local and metastatic	1124	All ages	Not specified	15–39 years (ref)	483	Cancer‐specific survival	Ref			Not reported			F	[17]
									0–14 years	327		HR = 1.00	0.70–1.43	Not reported					
									40–64 years	192		HR = 1.58	1.11–2.24	Not reported					
									≥ 65 years	122		HR = 3.26	2.29–4.64	Not reported					
Evenhuis	Cancers (Basel)	2021	Single center	Retrospective	Local and metastatic	402	3–82	MAP	0–16 (ref)	114	Event free survival	Ref			Ref			G	[18]
									16–40	218		HR = 1.50	1.07–2.11	< 0.05	HR = 1.31	0.89–1.94	0.17		
									≥ 40 years	70		HR = 1.71	1.09–2.67	< 0.05	HR = 1.33	0.80–2.19	0.27		
Lee	Pediatric Blood & Cancer	2009	Single center	Retrospective	Localized	288	All ages	MAPIB	< 12 + 15–39 years (ref)	203	Event‐free survival	Ref			Not reported			H	[19]
									12–15 years	69		HR = 1.93	1.26–2.69	0.002					
									≥ 40 years	16		HR = 1.96	1.03–3.74	0.04					
*Sex*																			
Smeland	European Journal of Cancer	2019	Multi center	Prospective	Local and metastatic	2186	< 40	MAP+/−IFNα/MAPIE	Female (ref) versus male	761/1106	Event‐free survival	HR = 1.2	1.03–1.39	0.017	HR = 1.40	1.16–1.70	0.001	A	[2]
Ottesen	JAAOS: Global Research & Reviews	2022	Multi center	Retrospective	Appendicular, local and metastatic	3435	< 23 to 62+	Not specified	Male (ref) versus female	1940/1495		Not reported			HR = 0.78	0.64–0.96	0.02	Not specified	[15]
Ottesen	JAAOS: Global Research & Reviews	2022	Multi center	Retrospective	Axial, local and metastatic	810	< 23 to 62+	Not specified	Male (ref) versus female	427/383		Not reported			HR = 0.80	0.71–0.90	< 0.001	Not specified	[15]
Duchman	Cancer Epidemiology	2015	Register based	Retrospective	Local and metastatic	2849	All ages	Not reported	Female (ref) versus male	1245/1604	Cause‐specific survival	HR = 1.1	1.00–1.31	< 0.05	Not reported			E	[16]
Fukushima	BMC Musculoskeletal Disorders	2018	Multi center	Retrospective	Local and metastatic	1124	All ages	Not specified	Male (ref) versus female	Not reported	Cancer‐specific survival	HR = 0.96	0.75–1.23	Not reported	Not reported			F	[17]
Tsuda	BMC Cancer	2018	Register based	Retrospective	Local and metastatic	760	≤ 40	MAPI	Male (ref) versus female	426/334	Disease‐specific survival	HR = 0.93	0.65–1.32	0.68	Not reported			Not specified	[20]
						173	41–64		Male (ref) versus female	93/80	Disease‐specific survival	HR = 0.75	0.42–1.33	0.32	Not reported				
						110	≥ 65		Male (ref) versus female	53/57	Disease‐specific survival	HR = 1.06	0.63–1.78	0.82	Not reported				
Evenhuis	Cancers (Basel)	2021	Single center	Retrospective	Local and metastatic	402	3–82	MAP	Male (ref) versus female	228/174	Event‐free survival	HR = 0.79	0.59–1.04	0.097	HR = 0.89	0.64–1.24	0.49	G	[18]
Xia	World Journal of Surgical Oncology	2016	Single center	Retrospective	Local and metastatic	359	19–69	Not reported	Male (ref) versus female	258/101	Progression‐free survival	HR = 1.02	0.77–1.35	0.89	HR = 1.06	0.78–1.44	0.70	I	[21]
Kim MS	Journal of Surgical Oncology	2008	Single center	Retrospective	Localized	331	3–40	Not specified	Female (ref) versus male	117/214	Metastasis‐free survival	HR = 1.20	0.78–1.83	0.41	Not reported			J	[22]
Durnali	Medical Oncology (Northwood, London, England)	2013	Multi center	Retrospective	Local and metastatic	240	13–74	AP/API/MAP/MAPI	Female (ref) versus male	87/153	Relapse‐free survival	HR = 1.75	0.52–5.94	0.36	HR = 1.22	0.29–5.20	0.79	K	[23]
Min D	Asia‐Pacific Journal of Clinical Oncology	2013	Single center	Retrospective	Local and metastatic	333	5–78	MAPI	Male (ref) versus female	211/122		Not reported			HR = 0.60	SE = 0.196	0.008	L	[24]
Buddingh	Pediatric Blood & Cancer	2009	Single center	Retrospective	Local and metastatic	56	< 40	AP+/−MBCyD/MAP	Male (ref) versus female	Not reported		Not reported			HR = 0.41	Not reported	0.05	Not specified	[25]

*Note:* Primary treatment: M: methotrexate, A: doxorubicin, P: cisplatin, IFNα: interferon alpha, I: ifosfamide, E: Etoposide, B: bleomycin, C: carboplatin, D: dactinomycin, Cy: cyclophosphamide. Variables in multivariate models: A. Stratified by study group and adjusted for tumor site, location within bone, pulmonary and non‐pulmonary metastases, sex, pathological fracture, age, relative tumor volume, histological response, surgical margins, and classification of sarcoma. B. Age, histologic subtype, surgery of primary tumor, tumor size, local extension, regional lymph node invasion, distant metastasis. C. Variables significant in univariate analyses were included in multivariate model: age, tumor volume, histologic response, ALP, treatment protocol, survival margin. D. Age, sex, tumor site, histologic subtype, ALP, LDH, tumor volume, chemotherapy protocol, type of surgery, histologic response. E. Age, sex, race, histologic subtype, metastatic disease, tumor location, size, socioeconomic variable. F. Age, sex, size, location, type of surgery, surgical margin. G. Age, tumor location, size, metastasis, surgical margin, response to chemotherapy, local recurrence of disease. H. Factors found to influence prognosis by univariate analysis were analyzed by multivariate Cox proportional hazard regression: Age, tumor length, tumor location, histologic response. I. Variables significant in univariate analyses were included in multivariate model: age, sex, stage, metastasis, neutrophil‐to‐lymphocyte ratio, platelet‐to‐lymphocyte ratio, post‐operative chemotherapy. J. Age, sex, AJCC stage, relative tumor size, tumor location, chondroblastic subtype, histologic response. K. Variables significant in univariate analyses were included in multivariate model: sex, metastasis, LDH, ALP, tumor margin, histologic response, type of chemotherapy. L. Variables significant in univariate analyses were included in multivariate model: sex, ALP, preop chemotherapy, postop chemotherapy, histologic response.

#### 3.1.2 Sex

Sex was evaluated in eleven studies (Table 2); six multicenter and five single‐center studies [2, 15, 16, 17, 18, 20, 21, 22, 23, 24, 25]. The median number of patients included was 380 (range: 56–3435 patients). The median male/female ratio was 1.3 (range: 0.92–2.55; data on sex ratio was missing for two studies) [17, 25]. Eight studies assessed sex in association with surrogate endpoints, of which two found a statistically significantly increased risk of worse outcome (10%–20%) for male patients. Seven studies evaluated the impact on OS, of which two showed a statistically significantly increased risk of death (20%–40%) for male patients.

### 3.2 Tumor's Features

#### 3.2.1 Metastatic Disease

Fifteen studies evaluated the presence of distant metastases at diagnosis and outcome (Table 3). Eight studies were multicenter (four prospective [2, 12, 26, 27], two retrospective [15, 23], two register‐based [16, 20]) and seven were single‐center retrospective studies [18, 25, 28, 29, 30, 31, 32]. The definition of metastatic disease was not specified in the studies. All fifteen studies consistently found that metastatic disease was associated with worse outcome compared to local disease, whichever endpoint was considered. The median hazard ratio (HR) was 3.04 (range: 2.34–7.2) for surrogate endpoints and 2.48 (range: 1.3–20.4) for OS [2, 15, 18, 23, 25, 26, 27, 29, 30, 31, 32]. One study focused on patients with lung metastases only and found a worse outcome for those with four or more lung metastases (4 vs. 1–3 lung metastases HR = 4.5, 95% CI 1.3–11.8). Kager et al. [33] found that having two or more distant metastases was associated with worse survival compared to having a single metastasis (HR = 2.3, 95% CI 1.2–4.3).

**TABLE 3:** Studies of tumor features as prognostic factors in newly diagnosed osteosarcoma. The studies are organized by prospective/retrospective data collection, prognostic factor categorization, reference value used, and number of included patients (*N*).

First author	Journal	Year	Multi/single center	Data collected prospectively/retrospectively	Population	*N*	Age span	Primary treatment	Strata	*N* per strata	Surrogate endpoints				Overall survival			Variables in multivariate model	Ref
												Point estimate	95% confidence interval	*p*	Point estimate	95% confidence interval	*p*		
*Metastases*																			
Smeland	European Journal of Cancer	2019	Multi center	Prospective	Local and metastatic	2186	< 40	MAP+/−IFNα/MAPIE	No (ref) versus lung metastases	1633/234	Event‐free survival	HR = 2.34	1.95–2.81	< 0.001	HR = 2.25	1.80–2.82	< 0.001	A	[2]
Tian S	Translational Oncology	2022	Multi center	Prospective	Local and metastatic	1199	Median 17, IQR 12–28	Not reported	No (ref) versus lung metastases	699/87	Cancer‐specific survival	HR = 3.52	2.56–4.84	< 0.001	Not reported			B	[12]
Smeland	European Journal of Cancer	2019	Multi center	Prospective	Local and metastatic	2186	< 40	MAP+/−IFNα/MAPIE	No (ref) versus non‐lung metastases	1809/58	Event‐free survival	HR = 2.38	1.38–2.73	< 0.001	HR = 2.79	1.92–4.04	< 0.001	A	[2]
Tian S	Translational Oncology	2022	Multi center	Prospective	Local and metastatic	1199	Median 17, IQR 12–28	Not reported	No (ref) versus non‐lung metastases	699/54	Cancer‐specific survival	HR = 2.98	1.99–4‐47	< 0.001	Not reported			B	[12]
Petrilli	Journal of Adolescent and Young Adult Oncology	2013	Multi center	Prospective	Local and metastatic	533	< 30	EpCI+/−M/CMAPI/MAP	Local (ref) versus metastatic disease	419/185	Event‐free survival	HR = 2.80	1.97–3.99	< 0.001	HR = 2.48	1.83–3.37	< 0.001	Not specified	[26]
Ozaki	Journal of Clinical Oncology	2003	Multi center	Prospective	Pelvic, local and metastatic	67	10–63	MAPIBCD	Local (ref) versus metastatic disease	52/15		Not reported			HR = 3.5	1.7–7.2	< 0.001	Not specified	[27]
Ottesen	JAAOS: Global Research & Reviews	2022	Multi center	Retrospective	Axial, local and metastatic	810	< 23 to 62+	Not specified	Local (ref) versus metastatic disease	661/149		Not reported			HR = 3.96	3.48–4.51	< 0.001	Not specified	[15]
Ottesen	JAAOS: Global Research & Reviews	2022	Multi center	Retrospective	Appendicular, local and metastatic	3435	< 23 to 62+	Not specified	Local (ref) versus metastatic disease	2815/620		Not reported			HR = 3.39	2.66–4.33	< 0.001	Not specified	[15]
Duchman	Cancer Epidemiology	2015	Register based	Retrospective	Local and metastatic	2849	All ages	Not reported	Local (ref) versus metastatic disease	2188/661	Cause‐specific survival	HR = 3.63	3.15–4.18	< 0.05	Not reported			C	[16]
Tsuda	BMC Cancer	2018	Register based	Retrospective	Local and metastatic	760	≤ 40	MAPI	Local (ref) versus metastatic disease	646/111	Disease‐specific survival	HR = 3.4	2.3–5.2	< 0.001	Not reported			Not specified	[20]
						173	41–64	MAPI	Local (ref) versus metastatic disease	139/34	Disease‐specific survival	HR = 3.04	1.63–5.69	< 0.001	Not reported				
						110	≥ 65	MAPI	Local (ref) versus metastatic disease	80/30	Disease‐specific survival	HR = 3.04	1.63–5.69	< 0.001	Not reported				
Ganguly	Frontiers in Oncology	2023	Single center	Retrospective	Local and metastatic	594	2–71	AP/APIE	Local (ref) versus metastatic disease	265/131	Event‐free survival	HR = 3.5	2.58–4.88	< 0.001	Not reported			D	[28]
Durnali	Medical Oncology (Northwood, London, England)	2013	Multi center	Retrospective	Local and metastatic	240	13–74	AP/API/MAP/MAPI	Local (ref) versus metastatic disease	191/49	Relapse‐free survival	HR = 7.2	1.78–29.4	0.006	HR = 7.67	1.61–36.6	0.01	E	[23]
Evenhuis	Cancers (Basel)	2021	Single center	Retrospective	Local and metastatic	402	3–82	MAP	Local (ref) versus metastatic disease	325/66	Event free survival	HR = 2.58	1.86–3.56	< 0.001	HR = 3.58	1.86–3.57	< 0.001	F	[18]
Basoli	Current Oncology	2023	Single center	Retrospective	Local and metastatic	210	11–16	Not specified	Local (ref) versus metastatic disease	159/51		Not reported			HR = 3.71	2.19–6.29	< 0.001	G	[29]
Yasin	Journal of Orthopaedic Surgery (Hong Kong)	2020	Single center	Retrospective	Local and metastatic	128	5–59	MAP	Local (ref) versus metastatic disease	50/78		Not reported			HR = 20.4	2.5–166.1	0.005	Not specified	[30]
Vasquez	Journal of Pediatric Hematology/Oncology	2017	Single center	Retrospective	Local and metastatic	55	< 18	MAPI	Local (ref) versus metastatic disease	19/36		Not reported			HR = 2.48	1.1–5.7	0.04	H	[31]
Buddingh	Pediatric Blood & Cancer	2009	Single center	Retrospective	Local and metastatic	56	< 40	AP+/−MBCyD/MAP	Local (ref) versus metastatic disease	Not reported		Not reported			HR = 1.3	Not reported	0.04	Not specified	[25]
Nataraj	Clinical and Translational Oncology	2015	Single center	Retrospective	Metastatic	102	8–48	APIE	1–3 (ref) versus > 3 lung metastases	32/56	Event‐free survival	HR = 2.7	1.0–7.3	0.04	HR = 4.5	1.3–11.8	0.05	I	[32]
Kager	Journal of Clinical Oncology	2003	Multi center	Retrospective	Metastatic	202	2–66	MAPIBCD	Lung/skip versus other metastases	9/21		Not reported			HR = 1.5	0.93–2.4	0.096	J	[33]
									One versus multiple organs metastases	160/42		Not reported			HR = 0.9	0.53–1.4	0.581		
									One versus multiple metastases	38/160		Not reported			HR = 2.3	1.2–4.3	0.012		
*Primary tumor size*																			
Smeland	European Journal of Cancer	2019	Multi center	Prospective	Local and metastatic	2186	< 40	MAP+/−IFNα/MAPIE	Small (ref) versus large (≥ 1/3 of involved bone)	851/680	Event‐free survival	HR = 1.29	1.09–1.51	0.002	HR = 1.21	0.99–1.49	0.06	A	[2]
Kim MS	Journal of Surgical Oncology	2008	Single center	Retrospective	Localized	331	3–40	Not specified	Small (< 25.5 cm^2^/m^2^; ref) versus large RTP (> 25.5 cm^2^/m^2^)	167/164	Metastasis‐free survival	HR = 2.09	1.38–3.17	0.001				K	[34]
Ferrari	Annals of Oncology	2001	Single center	Prospective	Localized	300	< 40	MAP/MAPI	Tumor volume > 150 (ref) versus ≤ 150 mL	132/165	Disease‐specific survival	HR = 0.6	0.4–0.9	< 0.03				L	[14]
Tian S	Translational Oncology	2022	Multi center	Prospective	Local and metastatic	1199	Median 17, IQR 12–28	Not reported	≤ 70 mm (ref)	232	Cancer‐specific survival	Ref			Not reported			B	[12]
									70–139 mm	431		HR = 1.52	1.08–2.12	0.015					
									> 139 mm	177		HR = 1.78	1.21–2.16	0.003					
Duchman	Cancer Epidemiology	2015	Register based	Retrospective	Local and metastatic	2849	All ages	Not reported	≤ 5 cm (ref)	326	Cause‐specific survival	Ref			Not reported			C	[16]
									> 5–10 cm	490		HR = 1.2	0.95–1.61	> 0.05					
									≥ 10 cm	842		HR = 1.6	1.28–2.13	< 0.05					
Lee	Pediatric Blood & Cancer	2009	Single center	Retrospective	Localized	288	< 40	MAPIB	≤ 6 cm (ref)	57	Event‐free survival	Ref			Not reported			M	[19]
									6–8 cm	62		HR = 2.59	1.10–6.13	0.03					
									> 8 cm	169		HR = 4.77	2.18–10.43	< 0.001					
Fukushima	BMC Musculoskeletal Disorders	2018	Multi center	Retrospective	Local and metastatic	1124	All ages	Not specified	≤ 8 cm (ref)	Not reported	Cancer‐specific survival	Ref			Not reported			N	[17]
									> 8–16 cm	Not reported		HR = 1.63	1.23–2.16	Not reported					
									> 16 cm	Not reported		HR = 2.84	1.86–4.35	Not reported					
Tsuda	BMC Cancer	2018	Register based	Retrospective	Local and metastatic	760	≤ 40	MAPI	≤ 8 cm (ref)	65	Disease‐specific survival	Ref			Not reported			Not specified	[20]
									> 8–16 cm	373		HR = 1.7	1.1–2.6	< 0.05					
									> 16 cm	296		HR = 2.1	1.1–3.9	< 0.05					
					Local and metastatic	173	41–64	MAPI	≤ 8 cm (ref)	70	Disease‐specific survival	Ref			Not reported			Not specified	
									> 8–16 cm	82		HR = 0.91	0.50–1.68	0.77					
									> 16 cm	15		HR = 1.50	0.56–3.96	0.43					
					Local and metastatic	110	≥ 65	MAPI	≤ 8 cm (ref)	36	Disease‐specific survival	Ref			Not reported			Not specified	
									> 8–16 cm	62		HR = 1.03	0.58–1.82	0.93					
									> 16 cm	9		HR = 2.84	1.16–6.97	0.02					
Jin Q	Journal of Cancer	2020	Single center	Retrospective	Localized	482	7–47	MAPI	< 8 cm (ref) versus ≥ 8 cm	204/482	Event‐free survival	HR = 1.8	1.27–2.56	< 0.05	Not reported			O	[35]
Evenhuis	Cancers (Basel)	2021	Single center	Retrospective	Local and metastatic	402	3–82	MAP	< 8 cm (ref) versus ≥ 8 cm	154/221	Event free survival	HR = 1.84	1.34–2.53	< 0.001	HR = 1.71	1.19–2.46	0.004	F	[18]
Wang	Oncotarget	2015	Single center	Retrospective	Local and metastatic	340	6–55	MAPI	≤ 8 cm (ref) versus > 8 cm	156/184	Lung metastasis‐free survival	HR = 2.61	1.72–3.97	< 0.001	HR = 1.81	1.15–2.85	0.01	P	[36]
Vasquez	Journal of Pediatric Hematology/Oncology	2017	Single center	Retrospective	Local and metastatic	55	< 18	MAPI	< 8 cm (ref) versus ≥ 8 cm	24/31		Not reported			HR = 1.30	0.2–9.2	0.8	H	[31]
Ozaki	Journal of Clinical Oncology	2003	Multi center	Prospective	Pelvic, local and metastatic	67	10–63	MAPIBCD	< 10 cm (ref) versus > 10 cm	13/45		Not reported			HR = 2.5	0.16–1.01	0.053	Not specified	[27]
Yasin	Journal of Orthopaedic Surgery (Hong Kong)	2020	Single center	Retrospective	Local and metastatic	128	5–59	MAP	< 10 cm (ref) versus > 10 cm	59/69		Not reported			HR = 1.10	0.51–2.32	0.82	Not specified	[30]
Ganguly	Frontiers in Oncology	2023	Single center	Retrospective	Local and metastatic	594	2–71	AP/APIE	≤ 10 cm (ref) versus > 10 cm	191/131	Event‐free survival	HR = 1.73	1.01–1.89	0.045	Not reported			PP	[28]
Petrilli	Journal of Adolescent and Young Adult Oncology	2013	Multi center	Prospective	Local and metastatic	533	< 30	EpCI+/−M/CMAPI/MAP	< 12 cm (ref) versus > 12 cm	226/184	Event‐free survival	HR = 1.18	0.82–1.68	0.37	HR = 1.27	0.94–1.70	0.12	Not specified	[26]
Han	World Journal of Surgical Oncology	2012	Single center	Retrospective	Local and metastatic	177	6–56	MAPI	< 6 cm (ref) versus ≥ 6 cm	75/102		Not reported			HR = 1.69	1.07–2.65	0.02	Not specified	[37]
*Primary tumor site*																			
Smeland	European Journal of Cancer	2019	Multi center	Prospective	Local and metastatic	2186	< 40	MAP+/−IFNα/MAPIE	Other limb (ref)	1562	Event‐free survival	Ref			Ref			A	[2]
									Proximal femur or humerus	234		HR = 1.50	1.22–1.84	< 0.001	HR = 1.67	1.30–2.14	< 0.001		
									Axial bone	71		HR = 1.53	1.10–2.13	0.01	HR = 1.85	1.25–2.72	0.002		
Petrilli	Journal of Adolescent and Young Adult Oncology	2013	Multi center	Prospective	Local and metastatic	533	< 30	EpCI+/−M/CMAPI/MAP	Tibia (ref)	160	Event‐free survival	Ref			Ref			Not specified	[26]
									Femur	318		HR = 1.51	1.00–2.29	0.047	HR = 1.58	1.13–2.23	0.007		
									Humerus	64		HR = 1.59	0.85–2.98	0.14	HR = 1.40	0.81–2.42	0.22		
									Other	62		HR = 1.37	0.65–2.78	0.40	HR = 1.53	0.7–2.97	0.21		
Fukushima	BMC Musculoskeletal Disorders	2018	Multi center	Retrospective	Local and metastatic	1124	All ages	Not specified	Arm (ref)	Not reported	Cancer‐specific survival	Ref			Not reported			N	[17]
									Leg	Not reported		HR = 1.19	0.72–1.98	Not reported					
									Trunk	Not reported		HR = 2.64	1.53–4.56	Not reported					
									Head and neck	Not reported		HR = 1.73	0.50–6.04	Not reported					
Duchman	Cancer Epidemiology	2015	Register based	Retrospective	Local and metastatic	2849	All ages	Not reported	Limb (ref) versus axial bone	2371/478	Cause‐specific survival	HR = 1.8	1.56–2.19	< 0.05	Not reported			C	[16]
Evenhuis	Cancers (Basel)	2021	Single center	Retrospective	Local and metastatic	402	3–82	MAP	Limb (ref) versus axial bone	372/30	Event free survival	HR = 1.28	0.77–2.12	> 0.05	HR = 0.87	0.45–1.69	0.68	F	[18]
Araki	Anticancer Research	2022	Single center	Retrospective	Localized	65	9–63	Not specified	Appendicular skeleton (ref) versus trunk	54/11	Metastasis‐free survival	HR = 1.9	0.77–4.9	0.16	Not reported			Q	[38]
Lee	Pediatric Blood & Cancer	2009	Single center	Retrospective	Localized	288	< 40	MAPIB	Distal femur, proximal tibia, fibula (ref)	236	Event‐free survival	Ref			Not reported			M	[19]
									Proximal humerus	28		HR = 1.95	1.16–3.25	0.01					
									Other locations	24		HR = 0.76	0.37–1.58	0.47					
Kim MS	Archives of Orthopaedic and Trauma Surgery	2009	Single center	Retrospective	Localized	347	3–39	Not specified	Other location (ref) versus proximal humerus	315/32	Metastasis‐free survival	HR = 1.90	1.19–3.05	0.007	HR = 2.01	1.17–3.48	0.01	R	[22]
*Pathological fracture*																			
Smeland	European Journal of Cancer	2019	Multi center	Prospective	Local and metastatic	2186	< 40	MAP+/−IFNα/ MAPIE	Pathological fracture no (ref) versus yes	1645/222	Event‐free survival	HR = 1.00	0.80–1.26	0.97	HR = 1.08	0.81–1.42	0.61	A	[2]
Kelley	Journal of Clinical Oncology	2022	Multi center	Retrospective	Localized	2847	2–71	Not specified	Pathological fracture no (ref) versus yes	2526/321	Event‐free survival	HR = 1.03	0.84–1.30	0.79	HR = 1.25	0.97–1.61	0.08	S	[39]
						2193	2–18	Not specified	Pathological fracture no (ref) versus yes	1951/242	Event‐free survival	HR = 0.97	0.74–1.26	0.81	HR = 1.07	0.79–1.45	0.66	S	
						654	19–71	Not specified	Pathological fracture no (ref) versus yes	575/79	Event‐free survival	HR = 1.25	0.78–2.01	0.36	HR = 1.89	1.15–3.13	0.013	S	
Puri	Journal of Surgical Oncology	2017	Single center	Retrospective	Local and metastatic	825	3–64	APIE	Pathological fracture no (ref) versus yes	521/31	Event‐free survival	HR = 1.3	0.8‐2.1	0.30	HR = 1.2	0.7–2.1	0.49	Not specified	[40]
Scully	Journal of Bone and Joint Surgery. American Volume	2002	Multi center	Retrospective	Localized	107	2–69	Not specified	Pathological fracture no (ref) versus yes	55/52	Local recurrence	HR = 6.2	2.6–28.1	0.005	Not shown		0.80	T	[41]
*Histologic subtype*																			
Smeland	European Journal of Cancer	2019	Multi center	Prospective	Local and metastatic	2186	< 40	MAP+/−IFNα/MAPIE	Chondroblastic (ref)	300	Event‐free survival	Ref			Ref			A	[2]
									Osteoblastic	1154		HR = 0.85	0.71–1.03	0.10	HR = 0.91	0.72–1.16	0.47		
									Other conventional	293		HR = 0.67	0.52–0.88	0.003	HR = 0.66	0.47–0.93	0.016		
									Telangiectatic	86		HR = 0.52	0.33–0.80	0.003	HR = 0.49	0.28–0.87	0.015		
									Small cell	10		HR = 1.48	0.60–3.64	0.39	HR = 1.47	0.53–4.06	0.46		
									High‐grade surface	24		HR = 0.44	0.19–0.99	0.047	HR = 0.28	0.07–1.14	0.076		
Ferrari	Annals of Oncology	2001	Single center	Prospective	Localized	300	< 40	MAP/MAPI	Not specified (ref)	25	Disease‐specific survival	Ref		0.03 global	Not reported			L	[14]
									Osteoblastic	195		HR = 1.4	0.7–2.9						
									Chondroblastic	33		HR = 1.0	0.4–2.4						
									Fibroblastic	22		HR = 0.5	0.1–1.5						
									Telangiectatic	25		HR = 0.6	0.2–1.6						
Duchman	Cancer Epidemiology	2015	Register based	Retrospective	Local and metastatic	2849	All ages	Not reported	Osteosarcoma NOS (ref)	2018	Cause‐specific survival	Ref			Not reported			C	[16]
									Chondroblastic	406		HR = 0.9	0.78–1.14	> 0.05					
									Fibroblastic	186		HR = 0.7	0.54–0.98	< 0.05					
									Telangiectatic	110		HR = 1.2	0.84–1.64	> 0.05					
									Small cell	31		HR = 1.1	0.64–2.02	> 0.05					
									Central	47		HR = 0.9	0.52–1.72	> 0.05					
									High‐grade surface	13		HR = 1.45	0.52–3.72	> 0.05					
									Paget	38		HR = 1.2	0.76–1.96	> 0.05					
Durnali	Medical Oncology (Northwood, London, England)	2013	Multi center	Retrospective	Local and metastatic	240	13–74	AP/API/MAP/MAPI	Osteoblastic	89		Not reported			Ref			E	[23]
									Chondroblastic	47					HR = 0.07	0.002–2.2	0.1		
									Fibroblastic	28					HR = 1.0	0.2–4.2	0.98		
									Telangiectatic	13					HR = 0.5	0.07–2.9	0.4		
Kim MS	Archives of Orthopaedic and Trauma Surgery	2009	Single center	Retrospective	Localized	347	3–39	Not specified	Osteoblastic (ref) versus chondroblastic	296/29	Metastatic‐free survival	HR = 1.05	0.59–1.87	0.87	HR = 0.84	0.40–1.74	0.63	R	[22]
*P‐glycoprotein expression*																			
Serra	International Journal of Oncology	2006	Multi center	Prospective	Localized	96	< 40	MAPI	P‐glycoprotein negative (ref) versus positive	41/53	Event‐free survival	HR = 3.4	1.4–7.9	0.005	HR = 4.7	1.4–16.3	0.01	U	[42]
Serra	Journal of Clinical Oncology	2003	Single center	Prospective	Localized	149	< 40	MAP/MAPI	P‐glycoprotein negative (ref) versus positive	102/47	Event‐free survival	HR = 3.4	1.9–6.0	< 0.0001	Not reported			V	[43]
Hornicek	Clinical Orthopaedics and Related Research	2000	Single center	Retrospective	Local and metastatic	33	7–65	MAP	P‐glycoprotein negative (ref) versus positive	18/15		Not reported			HR = 4	Not reported	“Significant”	X	[44]
Schwartz	Journal of Clinical Oncology	2007	Multi center	Retrospective	Localized	272	< 30	MAP	P‐glycoprotein negative (ref) versus positive	Not reported	Event‐free survival	HR = 1.00	0.58–1.80	> 0.05	Not reported			Y	[45]
Wunder	Journal of Clinical Oncology	2000	Multi center	Retrospective	Localized	123	4–70	AP/MAP/MAPI	MDR1 RNA expression low versus medium versus high	43/36/44	Disease‐free survival	HR = 1.01	0.68–1.50	0.97	Not reported			Z	[46]
*Tumor RNA signature*																			
Marchais	Cancer Research	2022	Multi center	Retrospective	Local and metastatic	79	< 50	MEI/APIAI	G1 (ref) versus G2	discovery cohort		Not reported			HR = 6.3	1.7–24.1	0.007	AA	[47]
						82			G1 versus G2	validation cohort					KM G1 better survival than G2		0.0004 log‐rank	Univariate	
						96			G1 versus G2	validation cohort					KM G1 better survival than G2		0.02 log‐rank	Univariate	

*Note:* Primary treatment: M: methotrexate, A: doxorubicin, P: cisplatin, IFNα: interferon alpha, I: ifosfamide, E: Etoposide, Ep: epirubicine, C: carboplatin, B: bleomycin, D: dactinomycin, Cy: cyclophosphamide. Variables in multivariate models: A. Stratified by study group and adjusted for tumor site, location within bone, pulmonary and non‐pulmonary metastases, sex, pathological fracture, age, relative tumor volume, histological response, surgical margins, and classification of sarcoma. B. Age, histologic subtype, surgery of primary tumor, tumor size, local extension, regional lymph node invasion, distant metastasis. C. Age, sex, race, histologic subtype, metastatic disease, tumor location, size, socioeconomic variable. D. Metastasis, size, pathologic fracture, ALP level, hemoglobin, neurovascular involvement. E. Variables significant in univariate analyses were included in multivariate model: sex, metastasis, LDH, ALP, tumor margin, histologic response, type of chemotherapy. F. Age, tumor location, size, metastasis, surgical margin, response to chemotherapy, local recurrence of disease. G. Metastasis, histologic response, ALP level. H. Histologic response, metastasis, size, type of surgery, neutrophil‐to‐lymphocyte ratio, platelet‐to‐lymphocyte ratio, pretreatment absolute lymphocyte count, absolute lymphocyte count at day 15. I. Factors with significance (*p* ≤ 0.10) in univariate analysis were taken into multivariate analysis: EFS: ALP, Metastatic site, number of lung metastases, uni/bilateral lung metastases, OS: ALP, metastatic site, surgical margin, number of lung metastases. J. Variables significant in univariate analyses were included in multivariate model: age, primary tumor location, single/multiple organ system metastases, lung/skip/other metastases, solitary/multiple metastases, incomplete surgery. K. Age, sex, AJCC stage, relative tumor size, tumor location, chondroblastic subtype, histologic response. L. Age, sex, tumor site, histologic subtype, ALP, LDH, tumor volume, chemotherapy protocol, type of surgery, histologic response. M. Factors found to influence prognosis by univariate analysis were analyzed by multivariate Cox proportional hazard regression: Age, tumor length, tumor location, histologic response. N. Age, sex, size, location, type of surgery, surgical margin. O. Variables significant in univariate analyses were included in multivariate model: tumor diameter, ALP, vascular invasion by MRI. P. Variables significant in univariate analyses were included in multivariate model: LMFS: size, stage, white blood cell count, neutrophil count, platelet count, LDH, ALP; OS: size, stage, neutrophil count, platelet count, LDH, ALP. PP. Variables significant in univariate analyses were included in multivariate model: tumor diameter, distant metastases, ALP. Q. Variables significant in univariate analyses were included in multivariate model: Platelet‐lymphocyte ratio, neutrophil count, LDH, tumor location. R. Variables significant in univariate analyses were included in multivariate model: stage, tumor growth pattern, tumor location, type of surgery, histologic response. S. Age, sex, pathological fracture, tumor site, localization within the bone, histologic subtype, primary metastases, relative tumor size, response to chemotherapy, total surgical remission, type of operation. T. Variables significant in univariate analyses were included in multivariate model: OS: pathological fracture, size, type of surgery, histologic response, local recurrence; Local recurrence: pathologic fracture, fracture union, fracture displacement. U. Variables significant in univariate analyses were included in multivariate model: Histologic subtype, P‐glycoprotein. V. P‐glycoprotein, age, tumor volume. X. Age, sex, tumor site, p‐glycoprotein. Y. Primary tumor site, LDH, timing of surgery, p‐glycoprotein. Z. Age, tumor size, site, histologic response, type of chemotherapy, MDR1. AA. Sex, histologic response, metastasis, tumor size, treatment, chemotherapy, pubertal status, G1/G2.

#### 3.2.2 Primary Tumor Size

Seventeen studies evaluated the prognostic role of tumor size (Table 3). Ten studies were single‐center experiences (one prospective, nine retrospective) [14, 18, 19, 28, 30, 31, 34, 35, 36, 37] and seven were multicenter studies (four prospective, one retrospective, and two register‐based) [2, 12, 16, 17, 20, 26, 27]. The median number of patients included was 402 (range: 55–2849). Thirteen studies (77%) included both patients with local and metastatic disease, while four studies only included patients with local disease. Two studies defined primary tumor size by volume (Relative Tumor Plane adjusted for body surface more or less than 25.5 cm^2^/m^2^ and tumor volume more or less than 150 mL, respectively) [14, 34]. The EURAMOS‐1 trial defined “large” tumors as those involving more than one third of the affected bone [2]. Fourteen studies (82%) defined tumor size by unidimensional measurements (cm or mm). Nine and five studies used two and three size categories, respectively. The most frequent cut‐off value was 8 cm (eight studies). Irrespective of the definition of tumor size and categories and endpoints used, a large primary tumor was associated with worse outcome compared to a small one, although not all associations were statistically significant.

#### 3.2.3 Primary Tumor Site

Eight studies evaluated primary tumor site and outcome (Table 3). Four studies were multicenter (two prospective, one retrospective, one register‐based) [2, 16, 17, 26] and four were retrospective single‐center experiences [18, 19, 22, 38]. The median number of included patients was 467 (range: 65–2849). The definitions of primary tumor sites, categories, and resectability differed between studies. Four studies found that axial bone location resulted in increased risk of surrogate endpoints compared to limb location, although the association was statistically significant in only two of the studies. For OS, one study found a significant difference between axial and limb location (HR = 1.85, 95% CI 1.25–2.72) [2] while another one did not (HR = 0.87, 95% CI 0.45–1.69) [18]. The definition of limb differed between the two studies. Petrilli et al. found that femur was associated with poorer outcome than tibia, while humerus was not associated [26]. Kim et al. found that proximal humerus site was associated with worse outcome compared to all other locations [22].

#### 3.2.4 Pathological Fracture

Pathological fracture at osteosarcoma diagnosis and outcome was assessed in four studies (one multicenter prospective [2], two multicenter retrospective [39, 41], one single‐center retrospective) [40] (Table 3). The median number of patients included was 1105 (range: 107–2847). Three studies evaluated both EFS and OS [2, 39, 41]. None of them found an association with EFS, while one study, Kelley et al. [39], found an association with OS among patients 19–71 years old but not among children. Scully et al. [41] found that pathological fracture was associated with an increased risk of local recurrence (HR = 6.2, 95% CI 2.6–28.1) but not with OS.

#### 3.2.5 Histologic Subtype

Five studies evaluated the prognostic role of histologic subtype (Table 3). Three were multicenter (two prospective, one register‐based) [2, 16, 23] and two were single‐center experiences (one prospective, one retrospective) [14, 22]. The studies used different categories of histologic subtypes and reference values in the regression model. The EURAMOS‐1 register cohort [2] demonstrated better EFS and OS for the telangiectatic (HR = 0.52, 95% CI 0.33–0.80 and HR = 0.49, 95% CI 0.28–0.87, respectively) and high‐grade surface subtypes (HR = 0.44, 95% CI 0.19–0.99 and HR = 0.66, 95% CI 0.47–0.93, respectively) compared to the chondroblastic subtype. In contrast, Ferrari et al. [14], Duchman et al. [16], and Durnali et al. [23] found no difference in outcome for the telangiectatic subtype compared to the “not specified”, “osteosarcoma NOS” and “osteoblastic” subtype, respectively. Durnali et al. [23] and Kim et al. [22] evaluated histologic subtype in association with OS without significant findings.

#### 3.2.6 P‐Glycoprotein Expression

Five studies described the prognostic impact of P‐glycoprotein (PgP): three multicenter studies (one prospective, two retrospective) [42, 46], and two single‐center experiences (one prospective, one retrospective) (Table 3) [43, 44, 45]. The median number of patients enrolled was 123 (range: 33–272). The method to evaluate PgP expression differed between studies (data not shown). Two studies found that increased PgP expression was associated with worse EFS [40, 43], while two studies found no association with EFS or DFS [42, 46]. Two studies found significantly worse OS among patients with PgP positive tumors relative to patients with PgP negative tumors [42, 44].

#### 3.2.7 RNA Signature

Through RNA sequencing of diagnostic osteosarcoma biopsies, Marchais et al. [47] identified two independent components that captured the tumor and microenvironment cell features, designated G1 and G2. Patients with G1 tumors had a better OS compared to patients with G2 tumors in multivariate analysis, including known prognostic factors such as sex, metastasis status, histologic response, and puberty status (HR = 6.3, 95% CI 1.7–24.1). The association was validated in two independent patient cohorts.

### 3.3 Serum and Plasma Markers

#### 3.3.1 Alkaline Phosphatase

Fourteen studies evaluated the prognostic role of alkaline phosphatase (ALP) level and outcome (Table 4). Two were retrospective multicenter trials [23, 50] and twelve were single‐center experiences (two prospective, ten retrospective) [13, 14, 24, 28, 29, 32, 35, 36, 37, 48, 49, 58]. The median number of patients included was 260 (range: 78–783). The patients had local disease in six studies, metastatic disease in two studies, and either local or metastatic disease in six studies. Eight of eleven studies found a statistically significant increased risk of surrogate endpoints in association with elevated/high ALP levels compared to normal/intermediate levels (median HR = 2, range 1.1–3.6). The three studies with non‐significant *p* values had point estimates in the same direction as those with significant *p* values. Eight of nine studies found worse OS among patients with high ALP levels compared to patients with normal/low ALP levels (median HR = 2.11, range 1.73–4.14) [24, 29, 32, 36, 37, 49, 50, 58].

**TABLE 4:** Studies of serum and plasma markers as prognostic factors in newly diagnosed osteosarcoma. The studies are organized by prospective/retrospective data collection, prognostic factor categorization, reference value used, and number of included patients (*N*).

First author	Journal	Year	Multi/single center	Data collected prospectively/retrospectively	Population	*N*	Age span	Primary treatment	Strata	*N* per strata	Surrogate endpoints				Overall survival			Variables in multivariate model	Ref
												Point estimate	95% confidence interval	*p*	Point estimate	95% confidence interval	*p*		
*Alkaline phosphatase (ALP)*																			
Ferrari	Annals of Oncology	2001	Single center	Prospective	Localized	300	< 40	MAP/MAPI	ALP elevated (ref) versus normal	141/159	Disease‐specific survival	HR = 0.9	0.6–1.3	0.6	Not reported			A	[14]
Bacci	Cancer	2006	Single center	Prospective	Localized	783	All ages	MAP/MAPBCD/MAPI/MAPIE	ALP normal (ref) versus elevated	492/291	Event‐free survival	HR = 2.1	1.6–2.7	< 0.0001	Not reported			B	[13]
Jin Q	Journal of Cancer	2020	Single center	Retrospective	Localized	482	< 50	MAPI	ALP normal (ref) versus elevated	186/296	Event‐free survival	HR = 1.45	1.00–2.11	Not reported	Not reported			C	[35]
Min D	Asia‐Pacific Journal of Clinical Oncology	2013	Single center	Retrospective	Local and metastatic	333	5–78	MAPI	ALP normal (ref) versus elevated	228/105		Not reported			HR = 2.02	SE = 0.217	0.001	D	[24]
Durnali	Medical Oncology (Northwood, London, England)	2013	Multi center	Retrospective	Local and metastatic	240	13–74	AP/API/MAP/MAPI	ALP normal (ref) versus elevated	103/108	Relapse‐free survival	HR = 2.05	0.76–5.52	0.16	HR = 0.39	0.07–1.95	0.25	E	[23]
Nataraj	Journal of Surgical Oncology	2015	Single center	Retrospective	Localized	237	2–66	APIE	ALP normal (ref) versus elevated	114/110		Not reported			HR = 2.1	1.1–3.9	0.03	F	[32]
Basoli	Current Oncology	2023	Single center	Retrospective	Local and metastatic	210	11–16	Not specified	ALP normal (ref) versus elevated	Not reported		Not reported			HR = 1.73	1.02–2.94	0.042	G	[29]
Kim	Cancer Medicine	2017	Single center	Retrospective	Local and metastatic	186	All ages	AP/API/Other	ALP normal (ref) versus elevated	79/94	Disease‐free survival	HR = 1.6	0.9–2.9	0.13	HR = 2.12	1.07–4.21	0.03	H	[48]
Nataraj	Clinical and Translational Oncology	2015	Single center	Retrospective	Metastatic	102	8–48	APIE	ALP normal (ref) versus elevated	46/52	Event‐free survival	HR = 2.5	1.4–4.3	< 0.001	HR = 2.2	1.2–4.3	0.01	I	[32]
Han	World Journal of Surgical Oncology	2012	Single center	Retrospective	Localized	177	6–56	MAPI	ALP normal (ref)	49	Disease‐free survival	Ref			Ref			Not specified	[37]
									ALP intermediate	76		HR = 1.5	0.8–2.8	0.16	HR = 1.46	0.81–2.65	0.21		
									ALP high	52		HR = 2.1	1.4–3.8	0.02	HR = 1.98	1.06–3.68	0.03		
Meyers	Journal of Clinical Oncology	1992	Single center	Retrospective	Localized	279	Not reported	MABCD/MAP	ALP intermediate (ref)	Not reported	Disease‐free survival	Ref			Not reported			J	[49]
									ALP low	Not reported		HR = 0.5	0.3–0.7	< 0.05					
									ALP high	Not reported		HR = 2	1.8–2.2	< 0.05					
Wang	Oncotarget	2015	Single center	Retrospective	Local and metastatic	454	6–55	MAPI	ALP low (ref) versus high	103/237	Lung metastasis‐free survival	HR = 1.74	1.08–2.78	0.02	HR = 4.14	1.91–8.99	< 0.001	K	[36]
Ganguly	Frontiers in Oncology	2023	Single center	Retrospective	Local and metastatic	594	2–71	AP/APIE	ALP ≤ 450 (ref) versus > 450 IU/L	189/176	Event‐free survival	HR = 1.5	1.10–2.05	0.01	Not reported			L	[28]
Mialou	Cancer	2005	Multi center	Retrospective	Metastatic	78	< 20	MAPIE/Other	ALP ≤ 500 (ref) versus > 500 IU/L	30/30	Event‐free survival	HR = 3.6	1.8–7.1	0.001	HR = 2.2	1.2–4.1	0.01	M	[50]
*Lactate dehydrogenase (LDH)*																			
Ferrari S	Annals of Oncology	2001	Single center	Prospective	Localized	300	< 40	MAP/MAPI	LDH high (ref) versus low	88/212	Disease‐specific survival	HR = 0.8	0.6–1.3	0.4	Not reported			A	[14]
Hu	Oncotarget	2017	Single center	Retrospective	Local and metastatic	106	7–53	MAP	LDH high (ref) versus low	26/80		Not reported			HR = 0.46	0.21–1.03	0.06	N	[51]
Kubo	Clinical Orthopaedics and Related Research	2015	Single center	Retrospective	Localized	37	10–55	MAP	LDH high (ref) versus low	Not reported		Not reported			HR = 0.16	0.02–1.58	0.117	O	[52]
Bacci	Tumori	2004	Single center	Retrospective	Localized	1222	All ages	10 different protocols	LDH low (ref) versus high	992/230	Disease‐free survival	HR = 1.8	1.2–2.8	0.003	Not reported			P	[53]
Durnali	Medical Oncology (Northwood, London, England)	2013	Multi center	Retrospective	Local and metastatic	240	13–74	AP/API/MAP/MAPI	LDH low (ref) versus high	101/81	Relapse‐free survival	HR = 3.36	1.31–8.60	0.01	HR = 9.01	2.18–37.3	0.002	E	[23]
Araki	Anticancer Research	2022	Single center	Retrospective	Localized	65	9–63	Not specified	LDH low (ref) versus high	22/43	Metastasis‐free survival	HR = 1.8	0.73–4.8	0.19	Not reported			Q	[38]
Meyers	Journal of Clinical Oncology	1992	Single center	Retrospective	Localized	279	Not reported	MABCD/MAP	LDH intermediate (ref)	Not reported	Disease‐free survival	Ref			Not reported			J	[49]
									LDH low	Not reported		HR = 0.4	0.04–0.8	< 0.05					
									LDH high	Not reported		HR = 1.5	1.3–1.8	< 0.05					
*Circulating tumor DNA*																			
Audinot*	Annals of Oncology	2024	Multi center	Retrospective	Local and metastatic	183	4–50	MEI/APIAI	Low (ref) versus high quantity	103/74	Progression‐free survival	HR = 2.2	1.8–3.40	< 0.001	HR = 5.53	1.42–4.50	0.002	R	[54]
Shulman	British Journal of Cancer	2018	Multi center	Retrospective	Localized	72	5–22	MAP/MAPIE	Detectable no (ref) versus yes	31/41	Event‐free survival	HR = 2.26	0.9–5.9	0.098	HR = 4.15	0.9–19.0	0.066	S	[55]
Lyskjær	European Journal of Cancerer	2022	Not specified	Retrospective	Local and metastatic	72	0–80	Not reported	Negative (ref) versus positive	43/29		Not reported			HR = 1.48	Not specified	0.36	T	[56]
*Neutrophil count*																			
Wang	Oncotarget	2015	Single center	Retrospective	Local and metastatic	454	6–55	MAPI	Neutrophil count < 6.4 (ref) versus ≥ 6.4 × 10^9^	263/77	Lung metastasis‐free survival	HR = 1.56	1.01–2.41	0.04	HR = 1.6	1.02–2.6	0.04	K	[36]
Araki	Anticancer Research	2022	Single center	Retrospective	Localized	65	9–63	Not specified	Neutrophil count > 4.0 (ref) versus ≤ 4.0 × 10^9^	29/36	Metastasis‐free survival	HR = 4.5	1.7–12‐3	< 0.01	Not reported			Q	[38]
*Neutrophil‐to‐lymphocyte ratio (NLR)*																			
Xia	World Journal of Surgical Oncology	2016	Single center	Retrospective	Local and metastatic	359	19–69	Not reported	NLR ≤ 3.4 (ref) versus > 3.4	Not reported	Progression‐free survival	HR = 1.65	1.3–2.2	< 0.05	HR = 1.80	1.35–2.41	< 0.05	U	[21]
Tian K	Cancer Management and Research	2022	Single center	Retrospective	Local and metastatic	87	10–67	Not specified	NLR ≤ 2.5 (ref) versus > 2.5	65/22		Not reported			HR = 3.65	1.07–12.5	0.039	V	[57]
Vasquez	Journal of Pediatric Hematology/Oncology	2017	Single center	Retrospective	Local and metastatic	55	< 18	MAPI	NLR ≤ 2 (ref) versus > 2	34/21		Not reported			HR = 2.3	1.1–5.3	0.046	X	[31]

*Note:* Primary treatment: M: methotrexate, A: doxorubicin, P: cisplatin, I: ifosfamide, B: bleomycin, C: carboplatin, D: dactinomycin, E: Etoposide. Variables in multivariate models:A. Age, sex, tumor site, histologic subtype, ALP, LDH, tumor volume, chemotherapy protocol, type of surgery, histologic response. B. Variables significant in univariate analyses were included in multivariate model: age, tumor volume, histologic response, ALP, treatment protocol, survival margin. C. Variables significant in univariate analyses were included in multivariate model: tumor diameter, ALP, vascular invasion by MRI. D. Variables significant in univariate analyses were included in multivariate model: sex, ALP, preop chemotherapy, postop chemotherapy, histologic response. E. Variables significant in univariate analyses were included in multivariate model: sex, metastasis, LDH, ALP, tumor margin, histologic response, type of chemotherapy. F. Variables significant in univariate analyses were included in multivariate model: performance status, type of surgery, ALP. G. Metastasis, histologic response, ALP level. H. Age, sex, AJCC stage, relative tumor size, tumor location, chondroblastic subtype, histologic response. I. Factors with significance (*p* ≤ 0.10) in univariate analysis were taken into multivariate analysis: EFS: ALP, Metastatic site, number of lung metastases, uni/bilateral lung metastases, OS: ALP, metastatic site, surgical margin, number of lung metastases. J. Tumor site, race, histologic response LDH, ALP. K. Variables significant in univariate analyses were included in multivariate model: LMFS: size, stage, white blood cell count, neutrophil count, platelet count, LDH, ALP; OS: size, stage, neutrophil count, platelet count, LDH, ALP. L. Metastasis, size, pathologic fracture, ALP level, hemoglobin, neurovascular involvement. M. Variables significant in univariate analyses were included in multivariate model: number of metastatic sites, lung metastases, bone metastases, resection of metastases, ALP. N. Variables significant in univariate analyses were included in multivariate model: age, sex, metastasis, LDH. O. Stage, LDH, histologic response, Glut‐1 expression. P. Variables significant in univariate analyses were included in multivariate model: chemotherapy protocol, type of surgery, ALP, LDH. Q. Variables significant in univariate analyses were included in multivariate model: Platelet‐lymphocyte ratio, neutrophil count, LDH, tumor location. R. Quantity of ctDNA, age, sex, metastasis. S. Detection of ctDNA, age, sex. T. Detection of ctDNA, metastasis. U. Variables significant in univariate analyses were included in multivariate model: age, sex, stage, metastasis, neutrophil‐to‐lymphocyte ratio, platelet‐to‐lymphocyte ratio, post‐operative chemotherapy. V. Variables significant in univariate analyses were included in multivariate model: metastasis, tumor volume, neutrophil‐to‐lymphocyte ratio, fibrinogen level. X. Histologic response, metastasis, size, type of surgery, neutrophil‐to‐lymphocyte ratio, platelet‐to‐lymphocyte ratio, pretreatment absolute lymphocyte count, absolute lymphocyte count at day 15.

* The study by Audinot et al. was added to the table for comparison but was not found in the search string, which included studies published in 2000–2023.

#### 3.3.2 Lactate Dehydrogenase

The prognostic role of lactate dehydrogenase (LDH) was evaluated in seven studies (one retrospective multicenter [23], one prospective single‐center [14], five retrospective single‐center) [38, 49, 51, 52, 53] (Table 4). The median number of patients enrolled was 240 (range: 37–1222). Three of five studies investigating surrogate endpoints found that high LDH levels were associated with worse outcomes compared to normal/low LDH levels. For OS, two of three studies found an association [23, 51, 52].

#### 3.3.3 Circulating Tumor DNA

Two retrospective studies evaluated the prognostic role of pretreatment circulating tumor DNA (ctDNA) levels (Table 4). Methylation‐based assays [56] and copy number alterations detection [55] were used for ctDNA detection. Patients with detectable ctDNA had a higher risk of adverse outcomes relative to patients with no detectable ctDNA, although the associations were not statistically significant.

#### 3.3.4 Neutrophil Count

Two retrospective single‐center studies evaluated the prognostic role of pretreatment neutrophil count in serum (Table 4) [36, 38]. The two studies used different cut‐off values to define high/low neutrophil count (6.4 × 10^9^ cells/mL [36] and 4 × 10^9^ cells/mL [38]) and different outcome measures. The two studies found contradicting associations.

#### 3.3.5 Neutrophil‐To‐Lymphocyte Ratio

Three retrospective single‐center studies evaluated pretreatment neutrophil‐to‐lymphocyte ratio (NLR) and outcome (Table 4) [21, 31, 57]. A median of 87 patients was included (range: 55–359). The studies used different cut‐off values to define low/high NLR (2, 2.5 and 3.4). All studies found that a high NLR was associated with worse OS compared to a low NLR.

## 4 Discussion

We conducted a systematic review to identify pretreatment prognostic factors in patients with newly diagnosed osteosarcoma to be used for stratifying patients or to be validated in the upcoming European clinical trial FOSTER‐CabOS. We found that previously established prognostic factors, age at diagnosis, the presence of metastases, primary tumor size, and primary tumor location (appendicular vs. axial), were consistently associated with outcome in identified studies. Although ALP level was consistently associated with prognosis, we could not firmly conclude that it was independent of other prognostic factors. The evidence for sex, histologic subtype, tumor PgP expression, and LDH level was less clear. We could not establish any new biological marker, but note that the RNA signature referred to as G1 and G2, and ctDNA detection at diagnosis are promising and should be further evaluated [47, 55, 56].

The comparison of results between individual studies was limited by differential categorization of the prognostic factor under investigation, the use of different reference values and covariates in the regression models, different endpoints, and heterogeneous patient populations (age range, local and/or metastatic disease, tumor resectability, and treatment given). This prevented the estimation of the true effect that each prognostic factor has on patient outcome. Nevertheless, it was possible to identify factors consistently associated with prognosis in the identified studies.

The youngest patients had the best and the oldest patients the worst outcomes in all identified studies. This was true for surrogate endpoints as well as for OS. Compared to children, the relative risk of an adverse outcome seems to be in the order of 1.3–1.8 in young adults, 1.6–2.2 among older adults, and 3.8–4 among elderly patients (Table 2). However, due to heterogeneity between the studies, it was not possible to estimate the true risk magnitude for specific age groups. Because different age categories were used, it was not possible to identify the most appropriate age groups for patient stratification in the clinical setting or clinical trials.

The impact of the patients' sex on osteosarcoma outcome remains controversial. In the three largest studies identified, males have a 10%–20% higher risk of adverse outcomes compared to females. Although the trend of the estimates is similar in most of the twelve studies identified, the difference was statistically significant in only five of twelve comparisons. Power might be an issue. However, only one study had less than 100 participants, and the outcome events were common (> 20%). In summary, the patients' sex may have no or a small impact on osteosarcoma outcome.

The presence of distant metastases at diagnosis was consistently associated with poor prognosis compared to localized disease in all identified studies, with a HR between 2 and 3.5 in most studies. One study compared different metastatic sites but found no clear difference in outcome [33]. Two studies found that survival decreased as the number of metastases increased [33, 58]. Noteworthy, the definition of metastases and indeterminate pulmonary lesions, and how indeterminate lesions were managed, was not explicitly stated in most studies and may have differed. Further studies of the impact of metastatic site and the number of metastases would be valuable.

Large primary tumors were associated with worse outcomes compared to small ones in most identified studies. There were no contradictory point estimates, although not all associations were statistically significant. Different definitions of tumor size were used, including the proportion of involved bone, tumor volume, and tumor diameter. Although most studies used tumor diameter to define tumor size, different categories were used. Therefore, the optimal cut‐off values for patient stratification were not apparent in the data. The data indicates a dose–response relationship, suggesting that tumor size should either be divided into several categories rather than dichotomized, or used as a continuous variable.

The impact of primary tumor site on prognosis is hard to discern, because identified studies used different site categories and reference values for comparison. For instance, some studies combine all extremity sites (limb, appendicular bone) while others separate different limbs or even different segments of the same bone (e.g., proximal vs. distal humerus or femur). Nevertheless, primary tumors located in axial bone or trunk were consistently associated with worse prognosis compared to extremity sites. Whether other locations, such as proximal or distal extremity sites, are associated with prognosis is not possible to discern. Limited data were available for craniofacial location, and comparisons between this rare osteosarcoma location and other sites are not reported.

The presence of pathological fracture at osteosarcoma diagnosis is a debated prognostic factor because of inconsistency in results between studies. Among four identified studies assessing pathological fracture and EFS and OS, one study found a statistically significant association with OS among adults, but not among children and not with EFS [39]. The two other studies found no association with EFS and OS [2, 40]. A fourth study found pathological fracture to be associated with local recurrence, but we found no study to confirm this [41]. Further studies are needed to define the role of pathological fracture for outcome in osteosarcoma patients.

The prognostic role of histologic subtype is not clear. The use of different categories and reference values in the regression models makes it hard to compare results between studies [2, 14, 16, 22, 23]. Most associations were not statistically significant. Moreover, classifying histologic subtype at diagnosis entails uncertainty, because it is evaluated on a tumor biopsy, which may not be representative of the whole tumor mass.

The prognostic role of PgP expression in the primary tumor was evaluated in five studies, of which three found an association with outcome [42, 43, 44] while two studies did not [45, 46]. There were important differences in the methodology used to evaluate PgP expression between the studies, making it difficult to compare the results. The controversy regarding the role of immunohistochemical staining for assessing PgP expression in osteosarcoma has been discussed elsewhere [3, 59]. The three studies that used immunochemistry assays used different methodologies, and one study did not follow the guidelines agreed upon at the consensus meeting for immunohistochemical detection of PgP in human tumor tissue samples [59]. Two meta‐analyses found that, when PgP was evaluated by immunohistochemistry following the aforementioned guidelines, increased expression at diagnosis was associated with unfavorable outcome [60, 61]. Based on these results, the Italian Sarcoma Group performed a first‐line clinical trial in which the PgP expression level at diagnosis guided the adjuvant treatment [3]. Nevertheless, some controversy remains, and further investigations are needed to confirm the role of PgP as a risk stratification variable in newly diagnosed osteosarcoma.

An RNA signature of the diagnostic tumor biopsies, referred to as G1 and G2, was associated with survival in a large homogeneously treated osteosarcoma patient population and two validation cohorts [47]. We found no other published study investigating the G1/G2 RNA signature, but an oral presentation at the 2023 Connective Tissue Oncology Society meeting reported consistent results in an independent pediatric osteosarcoma cohort [62]. These promising results motivate further studies to establish the G1/G2 RNA signature as a prognostic biomarker. Functional characterization associated G1 tumors with innate immunity and G2 tumors with angiogenic, osteoclastic, and adipogenic activities [47]. The tumor microenvironment plays a central role in osteosarcoma biology and potentially affects response to treatment and survival [63]. We found a few other studies evaluating tumor microenvironment markers for their prognostic role in osteosarcoma [64, 65, 66]. However, different immune‐infiltrate cells were analyzed in heterogeneous patient cohorts and with heterogeneous methodologies. Although intriguing, these data need to be validated in independent cohorts.

Our search identified only two studies investigating ctDNA as a pretreatment prognostic biomarker in newly diagnosed osteosarcoma, potentially because this research field is relatively recent in osteosarcoma. Albeit using different technologies, both studies found that detectable ctDNA at diagnosis was non‐significantly associated with inferior EFS and OS in multivariate analyses [55, 56]. Recently, in a period beyond the scope of our search, another study was published highlighting the promising role of this biomarker [54]. Audinot et al. analyzed a large cohort of osteosarcoma patients treated within the French prospective trial OS2006 and found that ctDNA level at diagnosis was an independent prognostic factor (PFS HR = 3.5, *p* = 0.002; OS HR = 3.51, *p* = 0.012) [54]. The collection of multiple blood samples to advance the research of ctDNA is encouraged [67] and should be incorporated in new clinical trials. This could validate these promising results and lead to agreement regarding ctDNA methodologies and cut‐off values.

A high/elevated ALP level in serum was consistently associated with adverse outcomes compared to a low/normal value, both among patients with localized and primary metastatic disease. ALP is an important metabolic factor suggestive of high tumor activity in bone cancers [58]. However, whether ALP is independent from measures of tumor burden (primary tumor size, presence of metastases, stage) or bone metastases is not clear. Among the six studies of patients with localized disease, three included tumor size in the multivariate model. Six studies included patients with both localized and metastatic disease [23, 24, 28, 29, 36, 48]. Min et al. found an association but did not adjust for the presence of metastasis [24]. Durnali et al. included metastases in the multivariate model and found no association [23]. Among the remaining four studies, three adjusted for the presence of metastases and one for primary tumor size and disease stage. All four found an association with overall survival, while three of four found an association with surrogate endpoints. These inconsistencies in findings and covariates in the multivariate model make it difficult to conclude that ALP is an independent prognostic factor.

LDH shows the same pattern of association as ALP, but with less evidence. The inconsistency of results and the fact that three of seven studies did not adjust for tumor burden and/or ALP in the multivariate model makes it hard to conclude that LDH is an independent prognostic marker.

NLR needs further validation as a biomarker for prognosis. Although there is some consistency in results, studies are few, study cohorts small, and different cut‐offs and endpoints were used, making it difficult to draw conclusions [21, 31, 57]. There was conflicting evidence for neutrophil count.

We used strict inclusion and exclusion criteria, which enabled us to identify observational studies of adequate scientific quality. We excluded studies reporting only univariate association tests, as these will be confounded by other prognostic factors and consequently of uncertain value. We further excluded studies of prognostic factors that were not validated in an independent cohort. While some of these factors may turn out to be valuable prognostic markers in the future, this cannot be determined at present. We did not pool estimated effects of individual prognostic factors because considerable heterogeneity between identified studies regarding patient populations, categorization of the prognostic variable, reference values, and outcome measures used would have made the pooled estimates difficult to interpret. We included studies published between 2000 and 2023 as we believe the patients included in these studies are representative of those we would include in clinical trials today in terms of diagnostic workup, staging procedures, oncological treatment, surgical techniques, and supportive care. Under this assumption, the identified studies are appropriate for identifying prognostic factors to use for patient stratification in current clinical trials.

In conclusion, we were able to confirm the prognostic value of age, tumor size, the presence of metastasis, and axial versus appendicular tumor location in newly diagnosed osteosarcoma. Further studies of these factors should focus on defining appropriate cut‐off values and specific patient populations. ALP and LDH need to be shown to be independent of established prognostic factors. The significance of patient sex, pathological fracture, and histologic subtype remain unclear. The G1/G2 RNA signature and ctDNA detection in plasma are promising biomarkers for prognosis that should be further evaluated. To advance osteosarcoma research, standardized biological samples collection is key [67]. Of equal importance are data harmonization initiatives such as HiBiSCUS [68], that enable large analytic osteosarcoma datasets. Such initiatives will accelerate the investigation and validation of prognostic factors and improve treatment stratification and outcomes in osteosarcoma.

## Author Contributions

**Elisa Tirtei:** conceptualization (lead), data curation (lead), investigation (equal), methodology (equal), visualization (lead), writing – original draft (lead), writing – review and editing (lead). **Sascha Wilk Michelsen:** conceptualization (equal), data curation (equal), investigation (equal), methodology (equal), writing – original draft (equal), writing – review and editing (equal). **Lianne M. Haveman:** conceptualization (equal), data curation (equal), investigation (equal), methodology (equal), writing – original draft (equal), writing – review and editing (equal). **Cristina Meazza:** conceptualization (equal), data curation (equal), investigation (equal), methodology (equal), writing – original draft (equal), writing – review and editing (equal). **Joana F. Oliveira:** data curation (equal), investigation (equal), writing – original draft (equal), writing – review and editing (equal). **Ayesha Rasool:** conceptualization (equal), data curation (equal), methodology (equal), writing – original draft (equal), writing – review and editing (equal). **Emanuela Palmerini:** conceptualization (equal), methodology (equal), writing – original draft (equal), writing – review and editing (equal). **Will Wilson:** conceptualization (equal), methodology (equal), writing – original draft (equal), writing – review and editing (equal). **Nathalie Gaspar:** conceptualization (equal), methodology (equal), writing – original draft (equal), writing – review and editing (equal). **Sandra J. Strauss:** conceptualization (equal), methodology (equal), writing – original draft (equal), writing – review and editing (equal). **Andri Papakonstantinou:** conceptualization (lead), investigation (equal), methodology (lead), writing – original draft (equal), writing – review and editing (equal). **Fredrik Baecklund:** conceptualization (equal), data curation (equal), investigation (equal), methodology (lead), supervision (lead), visualization (equal), writing – original draft (lead), writing – review and editing (lead).

## Conflicts of Interest

E.T., S.W.M., F.B., L.M.H., C.M., J.F.O., A.R., W.W.: No conflict of interest. E.P. has served on advisory boards for Daiichy Sankyo, Deciphera Pharmaceuticals, Eusa Pharma, and SynOx Therapeutics outside the submitted work. S.J.S. has served on advisory boards for Inhibrx, Awen Oncology, Tessellate Bio, and Bayer outside of the submitted work.

## Data Availability

Data sharing is not applicable to this article as no new data were created or analyzed in this study.
